# Salivary protein kinase C alpha and novel microRNAs as diagnostic and therapeutic resistance markers for oral squamous cell carcinoma in Indian cohorts

**DOI:** 10.3389/fmolb.2022.1106963

**Published:** 2023-01-10

**Authors:** Sheetanshu Saproo, Shashanka S. Sarkar, Vishakha Gautam, Chingmei W. Konyak, Gouri Dass, Arpita Karmakar, Mansi Sharma, Gaurav Ahuja, Anand Gupta, Juhi Tayal, Anurag Mehta, Srivatsava Naidu

**Affiliations:** ^1^ Department of Biomedical Engineering, Indian Institute of Technology Ropar, Rupnagar, Punjab, India; ^2^ Department of Computational Biology, Indraprastha Institute of Information Technology- Delhi (IIIT-Delhi), New Delhi, India; ^3^ Department of Dentistry, Government Medical College and Hospital, Chandigarh, India; ^4^ Rajiv Gandhi Cancer Institute and Research Centre, New Delhi, India

**Keywords:** non-invasive diagnostics, salivary RNAs, oral microbiota, oral squamous cell carcinoma (OSCC), liquid biospsy

## Abstract

Oral squamous cell carcinoma (OSCC) is the second leading cause of cancer-related morbidity and mortality in India. Tobacco, alcohol, poor oral hygiene, and socio-economic factors remain causative for this high prevalence. Identification of non-invasive diagnostic markers tailored for Indian population can facilitate mass screening to reduce overall disease burden. Saliva offers non-invasive sampling and hosts a plethora of markers for OSCC diagnosis. Here, to capture the OSCC-specific salivary RNA markers suitable for Indian population, we performed RNA-sequencing of saliva from OSCC patients (*n* = 9) and normal controls (*n* = 5). Differential gene expression analysis detected an array of salivary RNAs including mRNAs, long non-coding RNAs, transfer-RNAs, and microRNAs specific to OSCC. Computational analysis and functional predictions identified protein kinase c alpha (PRKCA), miR-6087, miR-449b-5p, miR-3656, miR-326, miR-146b-5p, and miR-497-5p as potential salivary indicators of OSCC. Notably, higher expression of PRKCA, miR-6087 and miR-449b-5p were found to be associated with therapeutic resistance and poor survival, indicating their prognostic potential. In addition, sequencing reads that did not map to the human genome, showed alignments with microbial reference genomes. Metagenomic and statistical analysis of these microbial reads revealed a remarkable microbial dysbiosis between OSCC patients and normal controls. Moreover, the differentially abundant microbial taxa showed a significant association with tumor promoting pathways including inflammation and oxidative stress. Summarily, we provide an integrated landscape of OSCC-specific salivary RNAs relevant to Indian population which can be instrumental in devising non-invasive diagnostics for OSCC.

## 1 Introduction

Oral squamous cell carcinoma (OSCC) accounts for about 90% of all oral cancers and ranks sixth in global cancer incidence (D'Cruz et al., 2018). India registers one-third of the global OSCC cases (Borse et al., 2020) and is the second leading cause of cancer-related morbidity and mortality in the country (https://gco.iarc.fr/). Despite significant progress in the therapies, the overall survival remains dismal at 20% (Veluthattil et al., 2019), and the projected incidence rates of OSCC in India are alarmingly high (Sharma et al., 2018). Tobacco (various forms), alcohol, betel quid, human papillomavirus, poor oral hygiene, malnutrition continue to be major etiological factors for OSCC (Rivera, 2015). Notably, OSCC prevalence is relatively higher in Indian rural communities, and low-income strata due to the over exposure to risk factors, lack of disease awareness and inadequate quality care (Su et al., 2021). Community level screening aided with non-invasive diagnosis can result in early therapeutic interventions, improved prognosis, and survival of OSCC patients.

Liquid biopsy (LB) is an emerging diagnostic paradigm which allows capturing tumor-shed diagnostic molecules enriched in bodily fluids such as urine, blood, or saliva non-invasively (De Rubis et al., 2019). LB coupled to high-throughput technologies identified a diverse repertoire of potential tumor markers including proteins, nucleic acids, metabolites, and circulating tumor cells in various bodily fluids (Alix-Panabieres and Pantel, 2021). Notably, the clinical utility of LB-derived biomarkers holds promise for diagnosis, disease stratification, and monitoring prognosis of a broad spectrum of cancers, non-invasively (Larson et al., 2021).

Saliva directly interacts with OSCC tumor therefore considered an ideal non-invasive source for OSCC biomarkers. Recent surge in the discovery of salivary biomarkers for OSCC assessment collectively reinforced the clinical importance of this approach (Nagler, 2009). Independent studies have identified potential salivary biomarkers including proteins (Yu et al., 2016), metabolites (Song et al., 2020), circulating tumor DNA (ctDNA) (Cristaldi et al., 2019), extracellular vesicles (Gai et al., 2018), and microRNAs (miRNAs) (Menini et al., 2021), that may have diagnostic utility for OSCC. Intriguingly, qualitative, and quantitative changes in the oral microbiota have been correlated to OSCC and appears to hold potential for OSCC diagnosis (Yang et al., 2018; Zhang et al., 2019).

Salivary profilings to detect OSCC-specific RNAs, and oral microbial analyses related to OSCC have been performed previously (Yakob et al., 2014). However, these studies predominantly represent western world and other ethnic groups outside India. Notably, studies indicated that cancer biomarkers are heterogenous and show intra and inter-ethnic variations (Jing et al., 2014; Sokolenko and Imyanitov, 2018; Hirko et al., 2022). Moreover, recent salivary proteomic studies (Csosz et al., 2017; Singh et al., 2020) to identify OSCC markers, highlighted the importance of population-specific diagnostic markers. Nevertheless, a comprehensive investigation of OSCC-specific salivary RNAs appropriate to Indian ethnicity remains undetermined. In this study, we performed RNA-sequencing (RNA-seq) on saliva samples of OSCC patients and normal controls. We report for the first time, a combined OSCC-specific salivary RNAs and oral microbial dysbiosis which can be instrumental in devising non-invasive diagnostics for OSCC.

## 2 Methods

### 2.1 Study cohorts, saliva collection and RNA isolation

Unstimulated saliva was collected from clinically confirmed OSCC patients (*n* = 9) and normal control (NC) (*n* = 5) with no clinical evidence of OSCC. Saliva collection and RNA isolation was conducted as described previously (Pandit et al., 2013). Briefly, subjects were requested to refrain from drinking or eating for at least an hour before the collection of saliva. Approximately, 5 mL of saliva was collected from subjects into pre-chilled RNase-free conical tubes, and 5 μL of 20 U/μL RNase inhibitor (Ambion) was added immediately to prevent RNA degradation. Saliva was centrifuged at 2,600 g for 15 min at 4°C to remove cells and other debris, and the collected cell free saliva (CFS) was used for total RNA isolation by the TRIzol method (Ambion). Before saliva collection, informed consent was obtained from subjects, and all related protocols were approved by the Institute review board, Rajiv Gandhi Cancer Institute and Research Centre, New Delhi. The sample collection complied the Declaration of Helsinki.

### 2.2 RNA-seq library preparation

RNA quantity and quality were analyzed on Bioanalyzer (Agilent 2,100). Small RNA-seq libraries were prepared using the Ion total RNA-seq v2.0 kit (Thermo Fisher Scientific) as per manufacturer’s protocol. To size-select the desired RNA products, Magnetic Bead Cleanup Module (Thermo Fisher Scientific) was used twice with the same sample. During the first bead binding, magnetic beads captured larger RNA species such as mRNA and lncRNA. During the second binding and with increased ethanol concentration, desired small RNA products (miRNA and other small RNA) in the supernatant re-bound to the magnetic beads. After washing the beads, the desired RNA products were eluted with pre-heated (80°C) Nuclease-free Water. Purified and size-selected RNA was converted into cDNA, and the barcodes and adaptors were ligated to the libraries. Library size distribution was verified by Bioanalyzer using a high-sensitivity DNA kit (Agilent) (Supplementary Figure S1). The libraries were diluted to 100 picomolar final concentration, and an equimolar pool was taken for clonal amplification. Template preparation for libraries on ionospheres was achieved using OneTouch 2 protocols (Thermo Fisher Scientific). The Ion 540 kit OT2 (Thermo Fisher Scientific) system in single-end mode with 200 bp chemistry was used for small RNA library sequencing.

### 2.3 RNA-seq data processing

Cutadapt (v1.8.1) algorithm was used to trim the 3′adaptor of the sequencing reads and assess the read quality. Sequencing reads were aligned to reference human genome (hg19) using Bowtie2 genome aligner (v2.2.5). Subsequently, mapped reads were screened for annotations using miRBase (v22) for miRNAs, and Gencode (v19) for other RNAs. Mapped sequencing reads were quantified using the Featurecount module (v1.4.6) of the R-package. For taxonomic classification of microbial reads, One Codex (Minot et al., 2015) algorithm was used. Total microbial diversity was calculated using the Shannon diversity index (read count ≥5, *p*-value ≤.05 was considered significant). The mean proportion of the sequencing reads aligned to bacterial taxa was calculated using Statistical analysis of taxonomic and functional profiles (STAMP) (v2.1.3) (Parks et al., 2014), *p*-value ≤.05. The differentially abundant taxa (*p* ≤ .05) were analyzed for associated metabolic pathways and gene ontologies (GO) using the Kyoto Encyclopedia of Genes and Genomes (KEGG) database (https://www.kegg.jp). The GOs associated with the metabolic functions were analyzed and visualized using Cytoscape (v3.8.2).

### 2.4 Differential gene expression analysis

We used expression profiles of CFS RNAs from 5 NC and 9 OSCC samples. Since the gene expression matrix of the bodily fluids is often noisy, we selected only those genes that possess >5 read counts in at least 2 samples. Differential gene expression analysis was done using the DESeq2 web application (https://yanli.shinyapps.io/DEApp) Differentially expressed (DE) genes were further filtered based on their log_2_ fold change (FC) ≥1 or ≤ -1, *p*-value <.05 for the downstream analysis. The volcano plots were built on the DESeq output using the GraphPad Prism 8.2.1.

### 2.5 Functional enrichment analysis

To predict the functions of DE mRNAs and miRNAs target genes, we performed pathway enrichment analysis (cancer only) using the PANTHER (http://www.pantherdb.org) with a false discovery rate (FDR) < .05. The predicted pathways and pathway-related genes were analyzed and visualized as a network using Cytoscape (Shannon et al., 2003) (v3.8.2). Target genes of the differentially abundant miRNAs were predicted using Target Scan (v7.2) with a cumulative weighted context ++ score cut-off of -.4. The gene set enrichment analysis (GSEA) was conducted using iDEP.951 web application (http://bioinformatics.sdstate.edu/idep/) using Molecular Signatures (MSig) and oncogenic signatures reference gene sets. The GSEA was visualized as a bubble plot using SRplots (www.bioinformatics.com.cn/plot_basic_gopathway_bubbleplot).

### 2.6 Expression, survival, and therapeutic response analysis of Differentially expressed genes

The expression profile of DE genes was analyzed using Head and Neck Squamous cell carcinoma (HNSCC) dataset available on The Cancer Genome Atlas (TCGA) portal. The protein expression data was retrieved from HNSCC dataset available on Proteomics Data Commons (PDC) portal. Survival analysis was performed using Kaplan-Meier method on Km plotter web tool (https://kmplot.com/analysis/). The correlation of drug response with respect to DE genes was determined by Receiver Operating Characterization (ROC) method (https://www.rocplot.org). The DE miRNAs-mediated drug resistance pathways were predicted and visualized using ROC plotter (https://www.rocplot.org/mirna/index).

### 2.7 Statistical analysis

The statistical significance of DE genes was calculated using nominal *p*-value in DESeq2 web application. The significance in the difference between Shannon diversities of NC and OSCC was calculated using Student’s t-test. The enrichment in microbial taxa and metabolic pathways was calculated using STAMP statistical tool.

## 3 Results

### 3.1 Salivary transcriptome profiling

**FIGURE 1:**
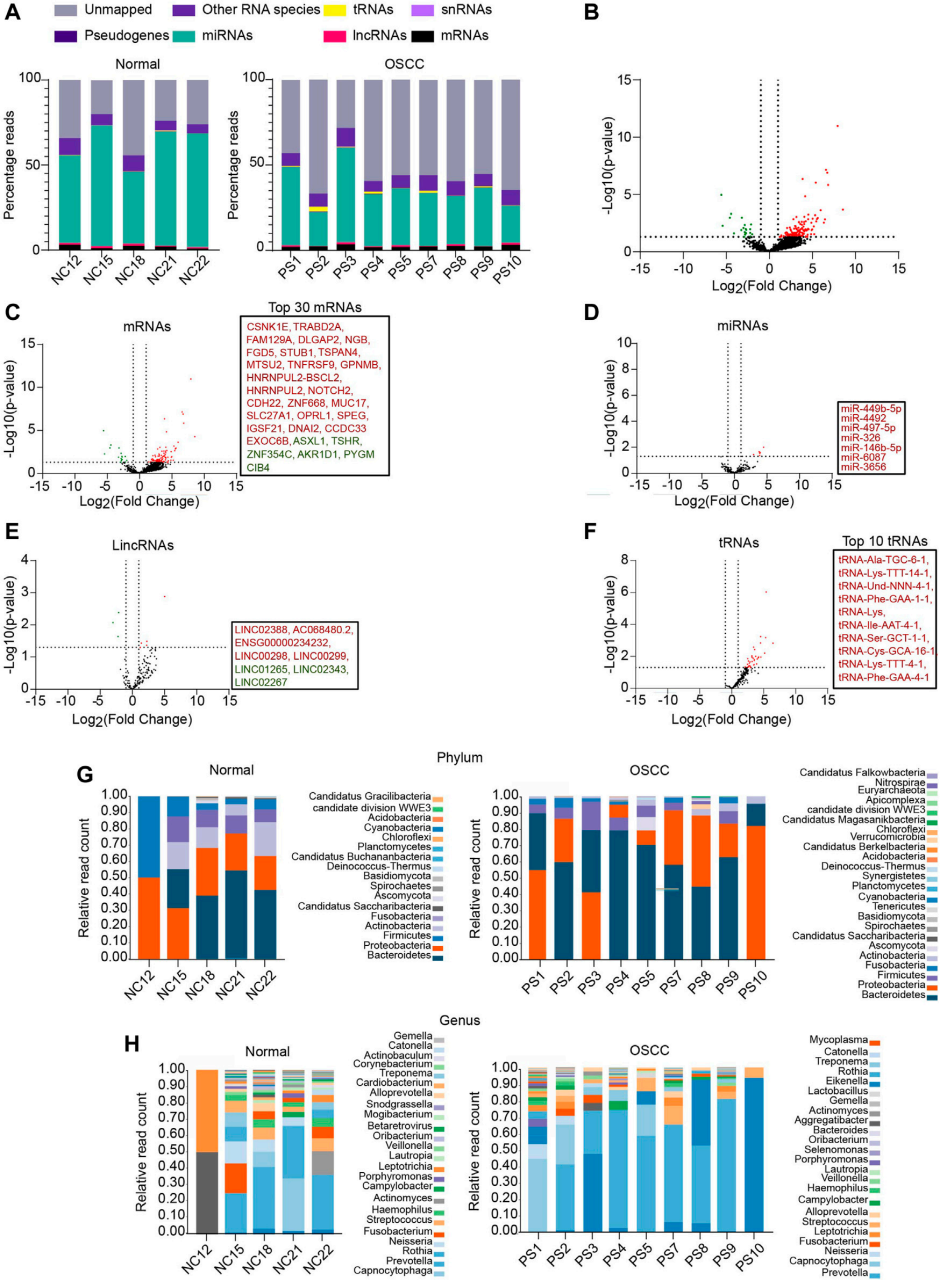
RNA profiling of salivary samples. **(A)** Read distribution of host salivary RNAs among normal and OSCC. **(B)** Volcano plots representing total altered transcripts in normal and OSCC (log_2_fold change ≥1 or ≤ -1, *p* ≤ .05). Volcano plots showing DE of **(C)** mRNAs, **(D)** miRNAs **(E)** lincRNAs, **(F)** tRNAs, respectively. Text boxes include names of top 30 mRNAs, all DE miRNAs, all DE lincRNAs, and top 10 tRNAs respectively. Relative read count of bacterial phyla **(G)** and genera **(H)** in normal and OSCC (cut off, ≥5 reads in at least 2 samples). NC = Normal control, PS = Patient sample.

To identify the salivary RNAs that segregate OSCC patients from normal, we performed small RNA-seq of the saliva sampled from OSCC patients and NC (the clinicopathological characteristics are described in Supplementary File S1). Approximately, 27 % of reads from the NC and 20 % from OSCC aligned to hg19 (Alignment statistics are detailed in Supplementary File S2). Among the mapped reads, RNA biotypes including mRNAs, miRNAs, long intergenic non-coding RNAs (lincRNAs), small nuclear RNAs (snRNAs), transfer RNAs (tRNAs), and pseudogenes were detected in both OSCC and NC (Figure 1A). DESeq analysis of the sequencing data yielded a total of 166 RNAs that were DE between OSCC and NC, of which 145 were upregulated and 21 were downregulated, with a log_2_ FC ≥ 1 or ≤ 1, *p*-value <.05 (Figure 1B; Supplementary File S3). Individual DE analysis of the RNA biotypes revealed 113 mRNAs (96 upregulated, and 17 downregulated) between OSCC and NC, (Figure 1C, top 30 DE mRNAs are shown in box). In addition, 7 miRNAs were found to be upregulated in OSCC compared to NC (Figure 1D) and 8 novel lincRNAs (5 upregulated, and 3 downregulated) (Figure 1E) are DE in OSCC. Notably, 35 novel tRNAs were found to be upregulated in OSCC (Figure 1F, top 10 tRNAs are shown in box). Other RNA species did not show any significant DE (Data not shown).

Salivary transcriptomes are enriched with oral microbial RNAs (Ostheim et al., 2020). Therefore, to examine whether the sequencing reads unmapped to hg19 were of microbial origin, we aligned these sequencing reads to the microbial genomes on ONE CODEX microbial analysis platform. Approximately, 9.76% of reads from OSCC, and 3.29% of reads from NC were aligned to annotated microbial genomes. Metagenomic analysis revealed a diverse range of bacterial phyla (Figure 1G) and genera (Figure 1H) in OSCC and NC samples. A large proportion of reads remain unmapped, we speculate that these reads plausibly belong to the other uncharacterized metagenomes whose sequences are unavailable to date. Summarily, we report an array of salivary RNAs that significantly segregate OSCC from NC, and RNAs annotated to oral microbiome.

### 3.2 Functional analysis of differentially expressed mRNAs

**FIGURE 2:**
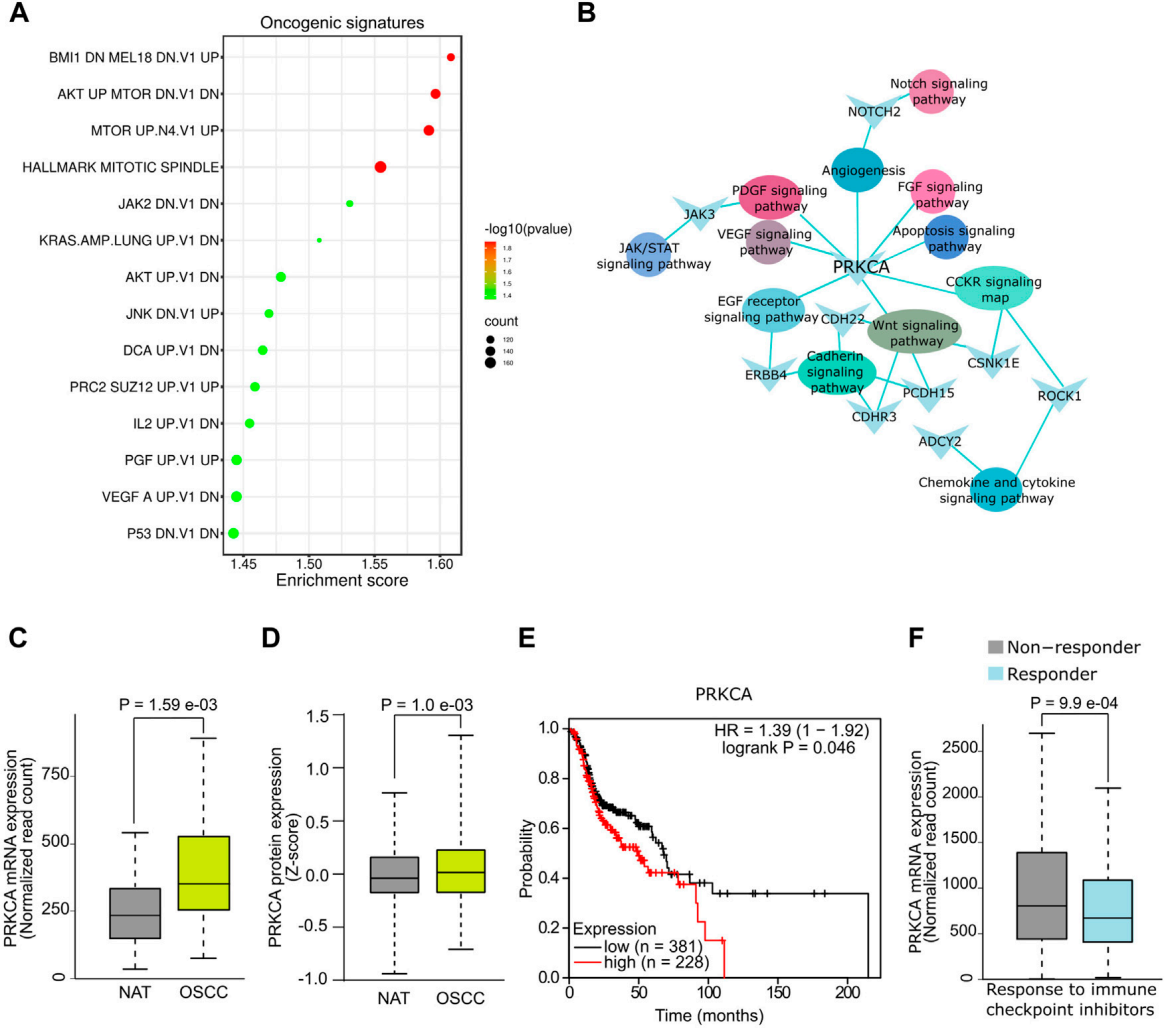
Functional analysis of salivary mRNAs. **(A)** GSEA showing association of DE mRNAs with oncogenic gene sets. **(B)** Functional networks showing DE hub mRNAs associated with multiple cancer pathways. DE of *PRKCA* at mRNA **(C)** and protein **(D)** levels in OSCC. **(E)** Survival plot showing reduced overall survival with high *PRKCA* expression. **(F)** Response of OSCC patients with high *PRKCA* expression towards immune checkpoint inhibitors.

The functional associations of DE mRNAs were examined by GSEA using gene sets of canonical oncogenic pathways available on MSig database. DE mRNAs were significantly (*p* < .05, FDR ≤.01) associated with multiple oncogenic signature pathways including BMl1, AKT, MTOR, KRAS, AKT, IL2, PGF and VEGF and p53 (Figure 2A, Supplementary File S4). In addition, GO analysis showed a significant association (*p* < .05) of mRNAs such as *PRKCA, NOTCH2, ZAK3, CDH22, ERBB4, CSNK1E, PCDH15, CDHR3, ROCK1*, and *ADCY2* with signaling pathways that are known to be dysregulated in OSCC (Figure 2B). Since *PRKCA* was found to be associated with multiple oncogenic pathways, we further analysed its diagnostic and prognostic relevance using OSCC TCGA data. Notably, expression analysis revealed a significant upregulation of *PRKCA* at mRNA (Figure 2C) and protein levels (Figure 2D) in OSCC tumors compared to adjacent normal control (NAT). Survival analysis indicated that OSCC patients with higher expression of *PRKCA* were relatively correlated with low survival (Figure 2E). PRKCA activates signaling pathways involved in chemotherapeutic drug resistance (He et al., 2022), this agrees with our GO analysis, therefore, we examined the plausible role of *PRKCA* in therapeutic resistance of OSCC. Importantly, patients with elevated expression of *PRKCA* showed resistance to immune checkpoint inhibitors including anti-PD1, anti-PDL1 and anti-CTLA-4 for OSCC therapy, Figure 2F shows a combined immune checkpoint therapy response with respect to PRKCA expression in OSCC patients. Taken together, salivary *PRKCA* expression could be a potential diagnostic, prognostic, and therapeutic marker of OSCC.

### 3.3 Differentially expressed miRNAs are associated with oral squamous cell carcinoma signaling pathways and multi-drug resistance

**FIGURE 3:**
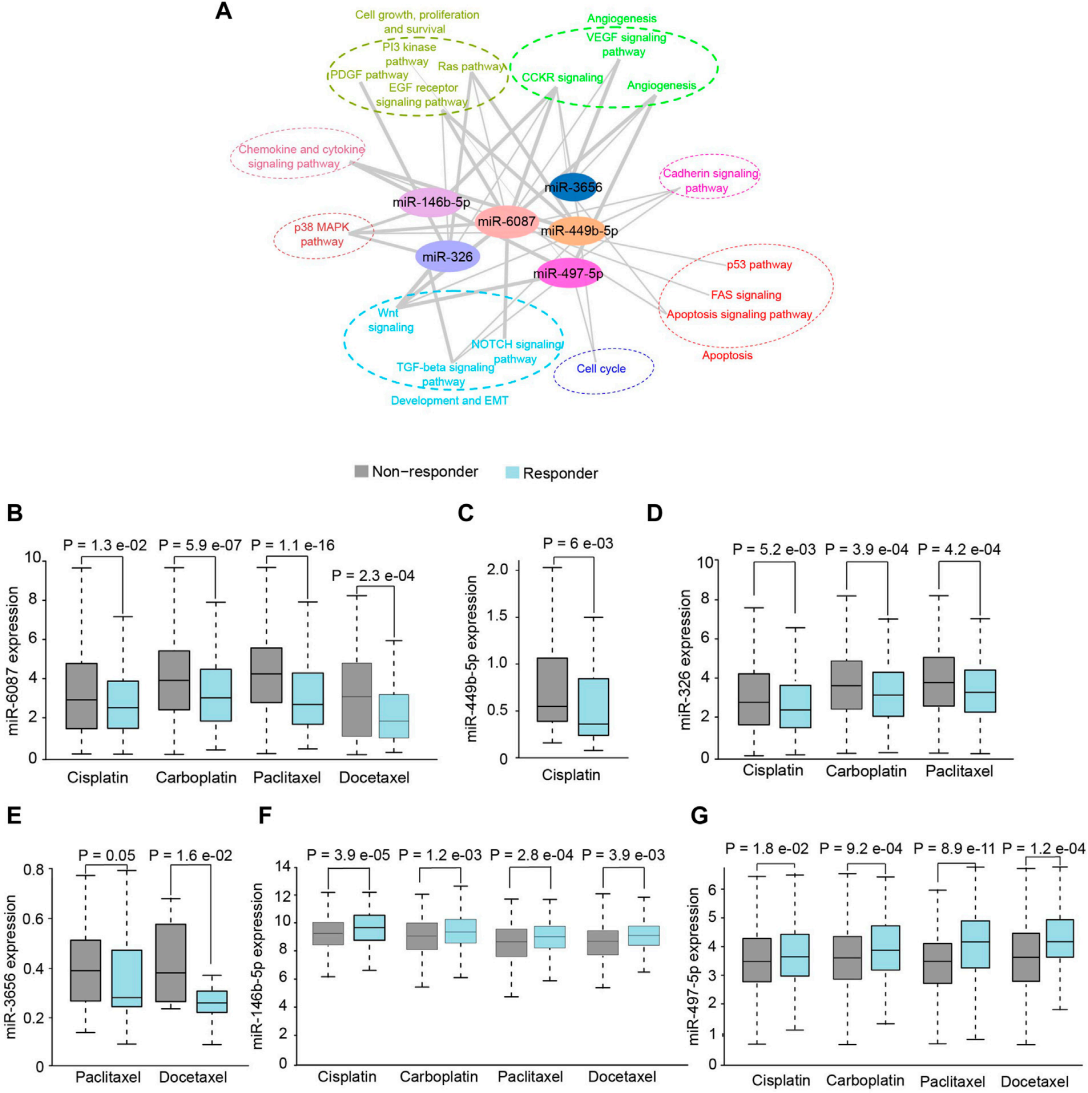
Functional analysis of salivary miRNAs. **(A)** Network analysis depicting association of differentially expressed miRNAs with cancer pathways. **(B–G)** Response of OSCC patients towards chemotherapeutics drugs with respect to higher expression of miR-6087, miR-449b-5p, miR-326, miR-3656, miR-146b-5p and miR-497-5p, respectively.

The putative targets of DE-miRNAs were predicted using the Targetscan (Supplementary File S5) and subjected to functional enrichment analysis. The putative targets of miR-6087, miR-449b-5p, miR-3656, miR-326, miR-146b-5p and miR-497-5p were found to be significantly (*p* < .05) enriched in hallmark pathways of cancer (Figure 3A). Since the targets of DE miRNAs were associated with therapeutic resistance including p38 MAPK, EMT, RAS-PI3K, we investigated their putative role in chemo-resistance. Notably, patients with higher expression of miR-6087, miR-449-5p, miR-326 and miR-3656 showed no-response to platinum and/or taxane based drugs, compared to patients with lower expression of these miRNAs (Figures 3B–E). Conversely, the expression of miR-146b-5p and miR-497-5p was relatively lower in non-responders to platinum and taxane compounds (Figures 3F,G). Collectively, miR-6087, miR-449b-5p, miR-3656, miR-326, miR-146b-5p, and miR-497-5p can serve as diagnostic and drug resistance markers for OSCC.

### 3.4 Oral microbial dysbiosis differentiated oral squamous cell carcinoma patients from normal controls

**FIGURE 4:**
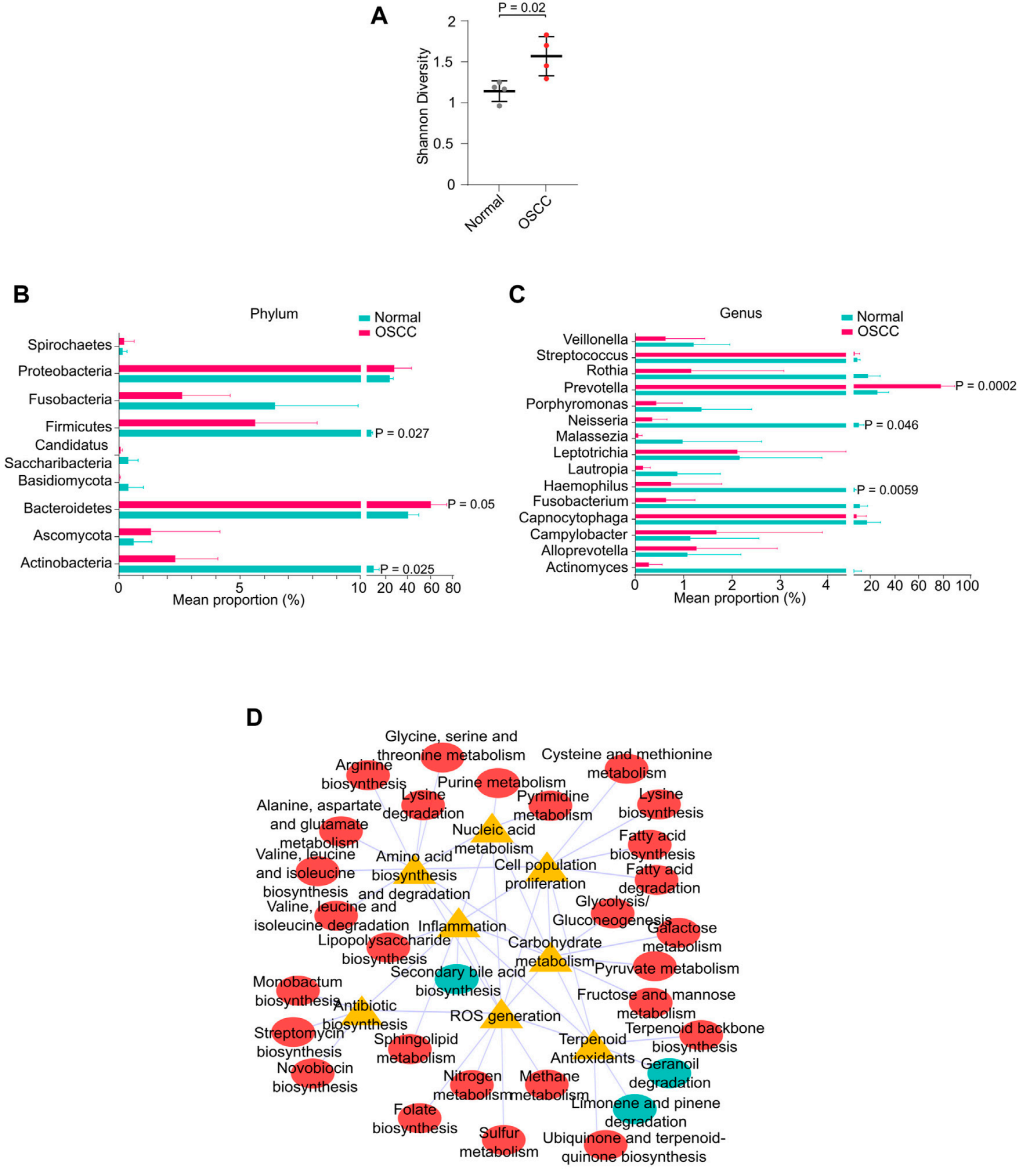
Oral microbial dysbiosis in OSCC. **(A)** Shannon diversity index showing microbial diversity between normal and OSCC. Mean proportion of microbial phyla **(B)** or genus **(C)** between normal and OSCC. **(D)** Interaction network between metabolic pathways and functional ontologies.

Recently, oral microbial dysbiosis is strongly associated with the incidence and progression of OSCC (Lee et al., 2017). Since a significant proportion of our sequencing reads mapped to microbial genomes, we measured the differences in microbial composition between OSCC and NC. Shannon diversity index showed a significantly higher microbial diversity in OSCC compared to NC (*p* < .05) (Figure 4A). Differential enrichment of microbial types by STAMP analysis revealed that phyla Firmicutes, and Actinobacteria were significantly enriched in NC (*p* < .05), and Bacteroidetes were significantly enriched in OSCC (*p* < .05) (Figure 4B). Among the 15 genera, *Prevotella* was significantly enriched in OSCC (*p* < .05), and *Neisseria* and *Haemophilus* were enriched in the NC group (Figure 4C). GO analysis of differentially abundant microbial taxa revealed associations with pathways including inflammation, ROS generation, cell proliferation, amino acid metabolism, nucleic acid metabolism, and carbohydrate metabolism suggesting a plausible role in carcinogenesis (Figure 4D). Summarily, our investigation not only identified prominent microbial dysbiosis between OSCC and NC, but also highlights the importance of analysing microbial sequences in salivary RNA-seq studies.

## 4 Discussion

The concept of LB is an emerging non-invasive or minimally invasive diagnostic paradigm of precision oncology. The cancer-specific molecular signatures detectable in bodily fluids faithfully surrogate various hallmarks of cancer, including tumor-heterogeneity (Snow et al., 2019; Liebs et al., 2021). The utility of saliva for OSCC diagnosis is gaining importance. In this study, we have identified OSCC-specific salivary RNAs of human and oral microbiome from Indian subjects. Notably, the combined detection of OSCC-specific RNAs and oral microbial dysbiosis from individual saliva samples minimizes the ambiguity in understanding the role of oral dysbiosis in OSCC.

GSEA revealed significant associations of DE mRNAs with oncogenic gene set signatures including BMI1, AKT, MTOR, JAK2, KRAS, JNK, PGF, VEGF, and P53. Notably, dysregulated BMI1 pathway has previously been associated with poor survival of OSCC patients (Hu et al., 2018). In addition, upregulated AKT/mTOR pathway activity has been attributed to chemo/radio-resistance and poor prognosis in OSCC (Harsha et al., 2020). Interestingly, the genes associated with the mutant p53 pathway were found to be enriched in our OSCC dataset. The upregulation of mutant p53 has previously been associated with lymph node metastasis in OSCC patients (Li et al., 2004). In addition, JAK pathway promotes the migration potential of OSCC cells (Chuang et al., 2014). Notably, among the upregulated mRNAs, Casein kinase 1 isoform epsilon (*CSNK1E*)*,* Tetraspanin-4 (*TSPAN4*)*, NOTCH2, NOTCH2NL,* and Protein kinase C Alpha (*PRKCA*) have previously been shown to have role in OSCC pathogenesis (Hirano et al., 2009; Lin et al., 2014; Porcheri et al., 2019; Xie et al., 2020). In addition, the higher expression of *TSPAN4* and *CSNK1E* in OSCC tumors has been shown to have diagnostic and prognostic potential (Hirano et al., 2009; Lin et al., 2014). Further, Notch signaling components has shown to be upregulated and oncogenic in OSCC (Porcheri et al., 2019). In line with these studies, it is highly plausible that the enrichment of these mRNAs in our study mirrors their expression in OSCC tumor.

Functional analysis of DE mRNAs revealed an association with oncogenic pathways of OSCC including, Wnt, cadherin, and CCKR. Upregulation of Wnt pathway components expression, and hyperactive Wnt signaling have been linked to an aggressive phenotype, poor therapeutic response, and metastasis of OSCC (Xie et al., 2020). Moreover, the crosstalk between *EGFR* and Wnt pathways appears to have diagnostic and prognostic value for OSCC (Lee et al., 2010). Therefore, the salivary abundance of mRNAs associated with these pathways can be potentially exploited for diagnosis and prognosis of OSCC. Notably, the salivary upregulation of *PRKCA* mimics its expression in OSCC tumors and correlated with low survival and therapy resistance. The upregulation of *PRKCA* has shown to have implications in OSCC (Ozaki-Honda et al., 2017), and attributed to chemoresistance by the phosphorylation of *BCL2* in leukemia (Jiffar et al., 2004). Our analysis revealed that the increased expression of *PRKCA* was associated with resistance to immune checkpoint inhibitors, which are currently being used as advanced therapeutics in OSCC. Immunotherapy has been reported to improve the overall survival in OSCC; however, the resistance to immunotherapy remains a major challenge (Dos Santos et al., 2021). Taken together, *PRKCA,* can serve as a potential salivary indicator for survival and immunotherapy efficacy of OSCC.

The DE of miRNAs in bodily fluids has shown to have immense potential as biomarkers for cancer diagnosis, stratification, and drug efficacy (Hayes et al., 2014). The predicted targets of miRNAs in our study were enriched in OSCC-relevant Wnt signaling, cadherin signaling chemokine, and cytokine signaling pathways. Among the significantly DE-miRNAs, miR-3656 and miR-4492 (Schneider et al., 2018), miR-497 (Hu et al., 2016), and miR-146b-5p (Scapoli et al., 2010) have previously been shown to be overexpressed in OSCC tumors and implicated as putative diagnostic markers. Therefore, it is highly plausible that the DE-miRNAs were sourced from primary tumor, and may hold diagnostic potential. The role of novel miRNAs, miR-6087, miR-449b-5p, and miR-326 reported here, in OSCC pathogenesis remains elusive. The aberrant expression of miRNAs has previously been associated with multi-drug resistance in OSCC (Li et al., 2019; Cheng et al., 2021). Notably, DE of the identified miRNAs significantly correlated with chemotherapeutic drug response. The drug resistance mechanism of miRNAs operates through targeting of drug-resistance genes (Si et al., 2019). Downregulation of *TP73*, putative target of miR-6087, has been reported to induce chemoresistance in multiple cancer types, independent of p53 (Irwin et al., 2003). Moreover, downregulation of pro-apoptotic gene *BAX*, putative target of miR-6087, has been reported to induce chemoresistance in a p53 dependent manner (McCurrach et al., 1997). Further, *MDM4*, a putative target of miR-6087 is reported to increase chemoresistance in breast cancer (Lam et al., 2010). The higher expression of miR-6087 in OSCC, can therefore, contribute to chemoresistance in both p53 dependent and independent manner. Our analysis revealed that increased expression of miR-146b-5p and miR-497-5p increases chemosensitivity in OSCC. In line with this, the putative targets of miR-146b-5p including *TRAF6* and *NOVA1* have been shown to increase chemoresistance in OSCC and hepatocellular carcinoma, respectively (Wu et al., 2018; Cao et al., 2021). Additionally, the putative targets of miR-497-5p including *PSMD7*, *CCND3*, *SMURF2*, and *WNT7A* increase chemoresistance in gastric cancer, acute myeloid leukemia, lung cancer and OSCC, respectively (Tian et al., 2018; Ray et al., 2020; Smith et al., 2021; Wang et al., 2021). Owing to these observations, it is highly plausible that miR-146b-5p and miR-497-5p may impart chemosensitivity in OSCC by targeting these mRNAs. Collectively, the identified miRNAs can further be explored for their potential as diagnostic markers and therapeutic targets in larger cohorts of OSCC.

The OSCC-specific dysbiosis of phyla Firmicutes, Bacteroidetes, Actinobacteria, and genus *Prevotella* identified in this study supports the previous studies (Lee et al., 2017; Zhang et al., 2019). Here we report a novel salivary dysbiosis of genus *Neisseria* and *Haemophilus*. The functional relevance of oral microbes in OSCC operates through various mechanisms, including the release of genotoxic substances, carcinogens, or onco-metabolites, inflammation, and ROS-mediated toxicity (Sami et al., 2020). Accordingly, the association of microbial types enriched in OSCC with GOs such as inflammation, reactive oxygen species generation, cell proliferation, and nucleic acid, amino acid metabolism strongly suggest their potential role in the carcinogenesis of OSCC.

In summary, the OSCC-specific RNA signatures and oral microbial dysbiosis identified in this study can have diagnostic utilities tailored for Indian population. However, further screening in large cohorts is necessary to evaluate the true diagnostic potential of the identified RNAs. Nevertheless, this study strongly advocates the LB coupled RNA-seq approach for developing RNA-based non-invasive diagnostics for OSCC and contributes to the emerging *Salivaomics*.

## Data Availability

The RNA-seq data presented in this study is available at GEO accession ID—GSE176077, and the analysis files are available in the Supplementary Material.
