# Supplementary material for: Salivary protein kinase C alpha and novel microRNAs as diagnostic and therapeutic resistance markers for oral squamous cell carcinoma in Indian cohorts
Source: Front Mol Biosci. 2023 Jan 10;9:1106963. doi: 10.3389/fmolb.2022.1106963 (PMC9871261; doi:10.3389/fmolb.2022.1106963)
Supplement: Supplementary file 3 [file DataSheet1.PDF]

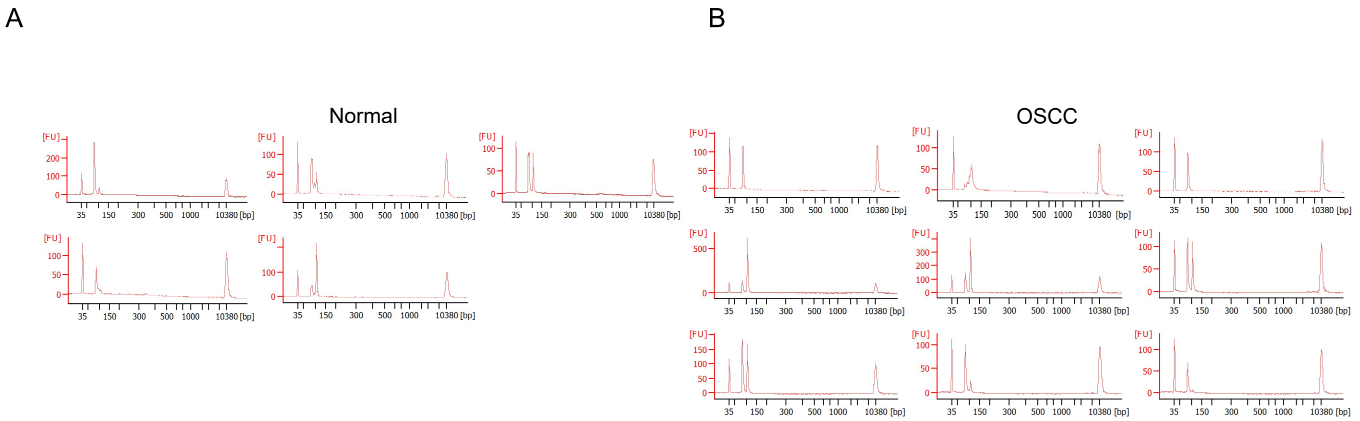

Supplementary Figure S1: Library profiles of normal and OSCC samples. (A), Bioanalyzer profiles for the libraries of RNA isolated from normal samples. (B), Bioanalyzer profiles for the libraries of RNA isolated from OSCC samples.
